# Diagnostic accuracy of urine dipstick testing for albumin-to-creatinine ratio and albuminuria: A systematic review and meta-analysis

**DOI:** 10.1016/j.heliyon.2021.e08253

**Published:** 2021-10-23

**Authors:** Jhonatan R. Mejia, Jose Ernesto Fernandez-Chinguel, Gandy Dolores-Maldonado, Naysha Becerra-Chauca, Sergio Goicochea-Lugo, Percy Herrera-Añazco, Jessica Hanae Zafra-Tanaka, Alvaro Taype-Rondan

**Affiliations:** a Universidad Nacional del Centro del Perú, Sociedad Científica de Estudiantes de Medicina del Centro, Huancayo, Peru; b Universidad San Martin de Porres, Chiclayo, Peru; c Instituto de Evaluación de Tecnologías en Salud e Investigación-IETSI, Lima, Peru; d Universidad Privada San Juan Bautista, Lima, Peru; e Universidad Científica del Sur, Escuela de Medicina, Lima, Peru; f Universidad San Ignacio de Loyola, Unidad de Investigación para la Generación y Síntesis de Evidencias en Salud, Lima, Peru

**Keywords:** Albuminuria, Sensitivity and specificity, Renal insufficiency, Chronic

## Abstract

**Background:**

The accuracy of urine dipsticks to detect increased albuminuria is uncertain. We aimed to assess the diagnostic accuracy of urine dipsticks for detecting albuminuria.

**Methods:**

A systematic review of studies that assessed the diagnostic accuracy of urine dipstick testing for detecting albuminuria has been conducted (using as reference standard the albuminuria in a 24-hour sample or the albumin-to-creatinine ratio) in Scopus, PubMed, and Google Scholar. The risk of bias of the included studies has been assessed using the Quality Assessment of Diagnostic Accuracy Studies-2 (QUADAS-2) tool. Whenever possible, we performed meta-analyses for sensitivity and specificity. The certainty of the evidence has also been assessed using the Grading of Recommendations Assessment, Development, and Evaluation methodology.

**Results:**

A total of 14 studies have been included in this review, having assessed all albumin-to-creatinine ratio (ACR) as assessed standard. Each study used different dipstick types. The resulting pooled sensitivity and specificity for each cutoff point were as follows: for ACR >30 mg/g (13 studies): 0.82 (95% confidence interval: 0.76–0.87) and 0.88 (0.83–0.91); for ACR 30–300 mg/g (7 studies): 0.72 (0.68–0.77) and 0.82 (0.76–0.89); and for ACR >300 mg/g (7 studies): 0.84 (0.71–0.90) and 0.97 (0.95–0.99), respectively. An overall high risk of bias, an important heterogeneity in all pooled analysis, and a very low certainty of the evidence have been found.

**Conclusions:**

Pooled sensitivity and specificity of urine dipsticks have been calculated for different ACR cutoff points. However, the dipstick types differed across studies, and the certainty of the evidence was very low. Thus, further well-designed studies are needed to reach more confident estimates and to assess accuracy differences across dipstick types.

**Registration:**

PROSPERO (CRD42019124637).

## 1 Introduction

For the diagnosis of chronic kidney disease (CKD), guidelines recommend performing an initial albuminuria testing using either the albumin-to-creatinine ratio (ACR) or the protein-to-creatinine ratio (PCR). When ACR and PCR are not available, some guidelines recommend the use of semiquantitative methods (urine dipsticks) that can measure albuminuria or express the result as ACR and the subsequent confirmation of the positive dipstick results with a quantitative laboratory test [1, 2]. However, no consensus regarding the use of urine dipsticks have been reached, and some guidelines do not support it [3].

Urine dipsticks represent a feasible point-of-care test that could be used in areas where other laboratory analyses are not available. However, its sensitivity for albuminuria detection is a bit concerning, since false negatives have been proposed to appear in the presence of ketones, glucose, blood, pigments, vitamins, or antibiotics [4, 5, 6], and a manual reading may incur operator-dependent faults [7].

Two previous systematic reviews have assessed the use of urine dipsticks for detecting albuminuria in the context of performing a CKD diagnosis. One of them performed its literature search in December 2013 and evaluated the diagnostic accuracy of point-of-care (POC) tests (either semiquantitative and quantitative) in people at risk of CKD, including nine studies that assessed the accuracy of urine dipsticks, reporting a pooled sensitivity and specificity of 0.76 and 0.93, respectively, for ACR >30 mg/g [8]. The other study is a Cochrane systematic review, which in September 2014 has searched for randomized controlled trials that assessed the effects of using urine dipstick testing for CKD diagnosis, and no other study was found [9].

Given the need for updated systematizations of the evidence to perform adequate decision-making, we aimed to perform a systematic review on the diagnostic accuracy of urine dipstick testing for detecting albuminuria.

## 2 Materials and methods

This systematic review was conducted following the Cochrane Handbook for Systematic Reviews of Diagnostic Test Accuracy and the Preferred reporting items for systematic review and meta-analysis of diagnostic test accuracy studies (PRISMA-DTA) reporting guidelines [10, 11]. The protocol was registered at PROSPERO (CRD42019124637, available at https://www.crd.york.ac.uk/prospero/display_record.php?RecordID=124637).

### 2.1 Data sources and searches

The search process had two steps: 1) Systematic searches have been performed in PubMed (via MEDLINE), Scopus, and Google Scholar in July 2020 (search terms are available in Table 1). 2) Furthermore, the references of each of the studies included in Step 1 were reviewed to find more eligible studies. The publication date has no restriction.

**Table 1:** Search strategy.

Search engine	Date	Term	Results
Pubmed	July 07, 2020	((“Reagent Strips”[Mesh] OR strip[tiab] OR strips[tiab] OR dipstick∗[tiab] OR point-of-care[TIAB] OR kit[TIAB]) AND (“Proteinuria”[Mesh] OR proteinuria[tiab] OR “Albuminuria”[Mesh] OR albuminuria[tiab] OR microalbuminuria[tiab]) AND (Sensitivity[tiab] OR Specificity[tiab] OR “Sensitivity and Specificity”[Mesh] OR “Predictive Value of Tests”[Mesh] OR “predictive value”[tiab] OR “Area Under Curve”[Mesh] OR “area under curve”[tiab] OR auc[tiab] OR “ROC Curve”[Mesh] OR roc[tiab] OR “receiver operating characteristic”[tiab] OR “receiver operating characteristics”[tiab] OR accuracy[tiab] OR predict∗[tiab])) NOT (letter [pt] OR editorial [pt] OR news [pt] OR historical article [pt] OR case reports [pt] OR letter[TI] OR comment∗[TI] OR animal∗[TI] OR “Animals, Laboratory”[Mesh] OR “Animal Experimentation”[Mesh] OR “Rodentia”[Mesh] OR rats[TI] OR rat[TI] OR mouse[TI] OR mice[TI] OR cat[ti] OR cats[ti] OR dog[ti] OR dogs[ti])	716
Scopus	July 07, 2020	TITLE-ABS-KEY ((“Reagent Strips” OR strip OR strips OR dipstick) AND (proteinuria OR ∗albuminuria) AND (Sensitivity OR Specificity OR “predictive value” OR “Area Under Curve” OR auc OR “ROC Curve” OR “receiver operating characteristic” OR accuracy OR predict∗)) AND NOT (KEY (“Laboratory animals” OR “Animal Experimentation” OR Rodentia) OR TITLE (animal∗ OR rodentia OR dog OR dogs OR cat OR cats OR mice OR mouse OR rat OR rats))	759
Google Scholar	July 07, 2020	Strip dipstick albuminuria sensitivity specificity	100

### 2.2 Studies selection and data extraction

Observational studies that met the following inclusion criteria have been included:

1. Evaluated the diagnostic accuracy of urine dipstick for albuminuria or ACR assessment.
2. Used a quantitative laboratory method for the assessment of the reference standard: either urine albumin (e.g., turbidimetry, nephelometry, or radioimmunoassay) or urine creatinine (e.g., Jaffe's Method).
3. If the reference standard was albuminuria, studies should have used 24-hour urinary samples.
4. Assessed the dipstick accuracy for any of the following cutoff values: ACR >30 mg/g or albuminuria >30 mg/l, ACR >300 mg/g or albuminuria >300 mg/l, or ACR between 30 and 300 mg/g or albuminuria between 30 and 300 mg/l, due to their clinical for CKD diagnosis or stratification according to clinical guidelines [1, 2].
5. Were written in English, Spanish, Portuguese, French, German, or Japanese.

After identifying the articles from the search strategy, duplicates were removed using the EndNote software. Then, each title and abstract were screened using the Rayyan QCRI application [12], and the potentially includable studies were assessed and reviewed in full text. Two independent reviewers conducted each step. Any discrepancy was discussed and solved by a third party.

### 2.3 Data extraction and quality assessment

For each study, information on each of the following main items was recorded: population, urine dipstick, urine specimen, reference standard, diagnostic accuracy measurements for each cutoff value, and funding. For the population, the age, the setting (general population, screening, primary care, and outpatient), the albuminuria prevalence (albuminuria >30 mg/dl or ACR >30 mg/g), and the number of patients have been collected. The following urine dipstick variables have also been collected: brand, type of albumin and creatinine measurement (colors or trace/+), and lecture (manually or automatically). Regarding the urine specimen, the time of collection (early morning, single random, or 24-hours), the number of measurements, and the number of samples have been collected. Concerning the reference standard test, the time of urine collection (early morning, single random, or 24-hours), the biochemical marker (albuminuria or ACR), and the laboratory method used to identify albumin and creatinine (turbidimetry, nephelometry, radioimmunoassay, Jaffe's, and enzymatic methods, etc.) have been recorded. Finally, the following diagnostic accuracy measurements have been collected: true positives, true negatives, false positives, and false negatives, which are needed to perform the meta-analyses. Two independent reviewers conducted each step of the data extraction. Any discrepancy was discussed and solved by a third party.

For the study quality assessment, two independent authors assessed the risk of bias using the revised tool for the quality assessment of diagnostic accuracy studies-2 (QUADAS-2) [13]. Discrepancies were solved by a consensus between the authors. The QUADAS-2 tool consists of four domains with two to three signaling questions for each domain. For the QUADAS-2 evaluation, criteria were used as reported elsewhere [13]. However, some specific criteria for this review have been established:

For the “index test” domain, the following values were considered as pre-specified thresholds for the index test: ACR >30 mg/g or albuminuria >30 mg/l, ACR >300 mg/g or albuminuria >300 mg/l, and ACR between 30 and 300 mg/g or albuminuria between 30 and 300 mg/l [1].

For the “reference standard” domain for creatinine, the test was considered as likely to correctly classify the target condition when isotope dilution mass spectrometry was used [14, 15, 16], while for protein and albumin, any technique has been considered (e.g., turbidimetry, nephelometry, and radioimmunoassay). In both cases, 24-hour urinary samples were considered as the reference standard [1, 2].

For the “flow and timing” domain, an appropriate interval between index test and reference standard was considered when the collection of the index and reference samples were performed simultaneously. Also, sample processing for both tests (index and reference) was necessary to be performed within the first 8 h after sample collection, or if any of those samples were processed after 8 h from collection, it was stated that such sample was preserved refrigerated [17, 18, 19].

Furthermore, a summary of the findings of our study was reported using the Grading of Recommendations Assessment, Development, and Evaluation (GRADE) methodology [20, 21].

### 2.4 Data synthesis and analysis

A meta-analyses was performed to obtain pooled sensitivities and specificities, along with their 95% confidence intervals (95% CIs). Random-effects models were performed given the high heterogeneity of the studies’ results. Heterogeneity was assessed using a chi-squared test and the I^2^ statistic. According to a recommendation stated on the PRISMA-DTA, a publication bias has not been evaluated due to the lack of a defined method that should be used in systematic reviews of diagnostic test accuracy studies [11]. Each analysis was performed in Stata v14.0 software (StataCorp LP, College Station, TX, USA).

Furthermore, the number of false-positive and false-negative results has been estimated, from a fictitious population of 1,000 individuals. To estimate the prevalence of ACR >30 mg/g, ACR between 30 and 300 mg/g, and ACR >300 mg/g, the median prevalence from the included studies that have measured all these three cutoff values has been calculated.

## 3 Results

### 3.1 Study selection

**Figure 1:**
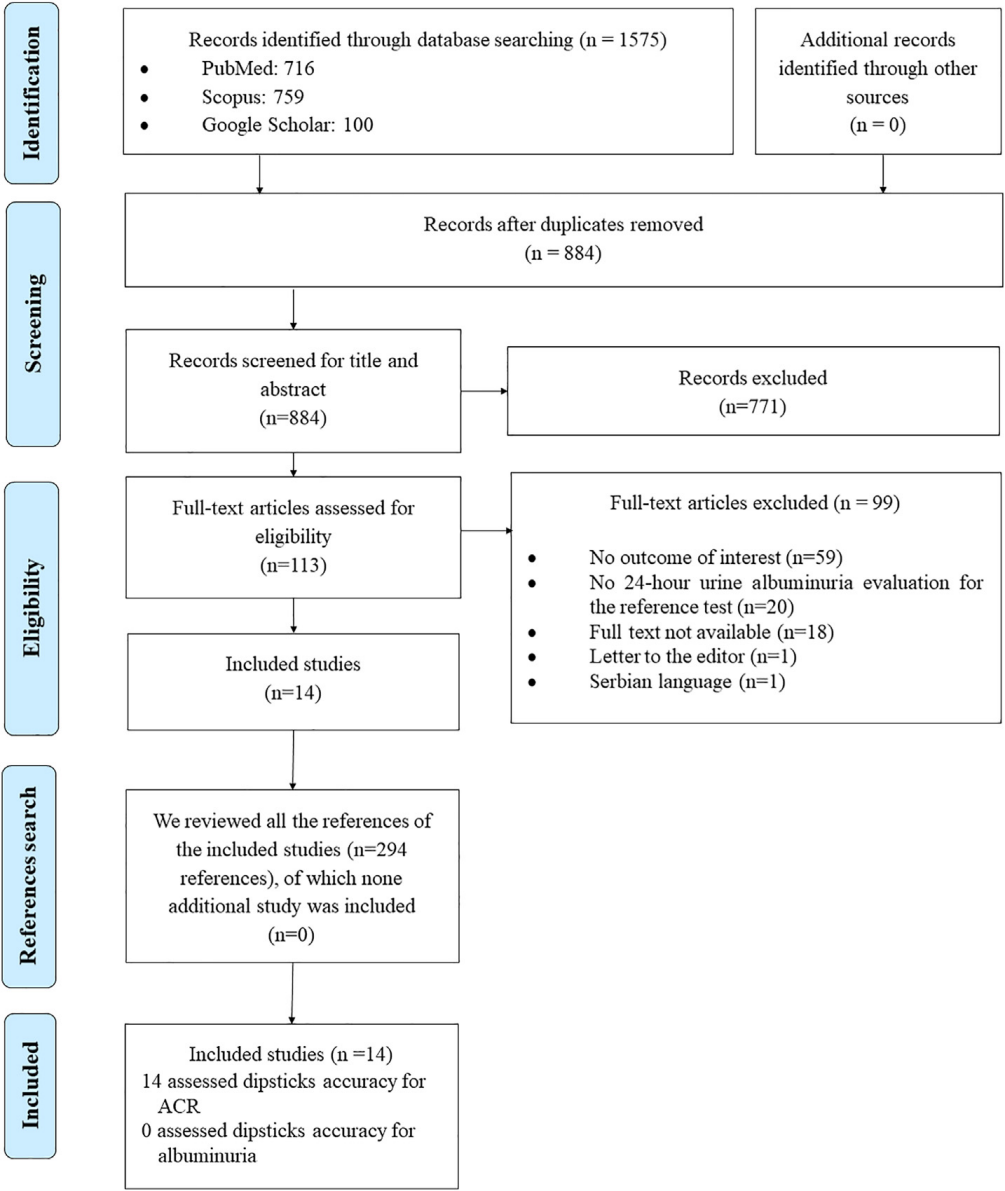
Flow diagram (study selection).

We found 1,575 records during database searching. After duplicate removal, 884 titles and abstracts have been screened, from which 113 underwent a full-text review, and finally 14 studies were included. Similarly, the 294 references of these manuscripts have been examined, and none have met the inclusion criteria. All 14 included studies have been assessed for the accuracy of urine dipsticks for ACR. No study was found that has assessed the accuracy of urine dipsticks for albuminuria directly (Figure 1 and Table 2).

**Table 2:** Studies that were evaluated in full text and were excluded.

N	Author	Year	Title	Exclusion reason
1	Sultana	2018	Dipstick Method versus Spot Urinary Protein Creatinine Ratio for Evaluation of Massive Proteinuria in Childhood Nephrotic Syndrome	No outcome of interest
2	Yanagisawa	2018	Prevalence of Chronic Kidney Disease and Poor Diagnostic Accuracy of Dipstick Proteinuria in Human Immunodeficiency Virus-Infected Individuals: A Multicenter Study in Japan	No outcome of interest
3	Ratnayake	2017	Screening for chronic kidney disease of uncertain etiology in Sri Lanka: usability of surrogate biomarkers over dipstick proteinuria	No outcome of interest
4	Koeda	2016	Comparison between urine albumin-to-creatinine ratio and urine protein dipstick testing for prevalence and ability to predict the risk for chronic kidney disease in the general population (Iwate-KENCO study): A prospective community-based cohort study	No outcome of interest
5	Chang	2016	The efficacy of semiquantitative urine protein-to-creatinine (P/C) ratio for the detection of significant proteinuria in urine specimens in health screening settings	No outcome of interest
6	Lopez De Leon	2015	Strong correlation between protein reagent strip and protein-to-creatinine ratio for detection of renal dysfunction in HIV-infected patients: a cross-sectional study	No outcome of interest
7	Lim	2014	Diagnostic accuracy of urine dipstick for proteinuria in older outpatients	No outcome of interest
8	Masimango	2014	Prevalence of microalbuminuria and diagnostic value of dipstick proteinuria in outpatients from HIV clinics in Bukavu, the Democratic Republic of Congo	No outcome of interest
9	Wahbeh	2014	Spot urine protein-to-creatinine ratio compared with 24-hour urinary protein in patients with kidney transplant	No outcome of interest
10	Kumar	2013	Comparison of urinary protein: Creatinine index and dipsticks for detection of microproteinuria in diabetes mellitus patients	No outcome of interest
11	Bello	2012	Multiple versus single and other estimates of baseline proteinuria status as predictors of adverse outcomes in the general population	No outcome of interest
12	Viana	2012	Prediction of cardiovascular events, diabetic nephropathy, and mortality by albumin concentration in a spot urine sample in patients with type 2 diabetes	No outcome of interest
13	Chotayaporn	2011	Comparison of proteinuria determination by urine dipstick, spot urine protein creatinine index, and urine protein 24 h in lupus patients	No outcome of interest
14	White	2011	Diagnostic accuracy of urine dipsticks for detection of albuminuria in the general community	No outcome of interest
15	Panek	2011	Screening for proteinuria in kidney transplant recipients	No outcome of interest
16	Lin	2011	The characteristics of new semiquantitative method for diagnosing proteinuria by using random urine samples	No outcome of interest
17	Collier	2009	A study of the relationship between albuminuria, proteinuria, and urinary reagent strips	No outcome of interest
18	Guy	2009	Diagnostic accuracy of the urinary albumin: creatinine ratio determined by the CLINITEK Microalbumin and DCA 2000 + for the rule-out of albuminuria in chronic kidney disease	No outcome of interest
19	Haysom	2009	Diagnostic accuracy of urine dipsticks for detecting albuminuria in indigenous and non-indigenous children in a community setting	No outcome of interest
20	Krol	2009	Early detection of chronic kidney disease: results of the PolNef study	No outcome of interest
21	Afolabi	2009	Prevalence of chronic kidney disease in a Nigerian family practice population	No outcome of interest
22	Biswas	2009	Quantitation of proteinuria in nephrotic syndrome by spot urine protein creatinine ratio estimation in children	No outcome of interest
23	Guy	2009	Use of a first-line urine protein-to-creatinine ratio strip test on random urines to rule out proteinuria in patients with chronic kidney disease	No outcome of interest
24	Siedner	2008	Diagnostic accuracy study of urine dipstick in relation to 24-hour measurement as a screening tool for proteinuria in lupus nephritis	No outcome of interest
25	Abo-Zenah	2008	Prevalence of increased albumin excretion rate in young Saudi adults	No outcome of interest
26	Garcia	2006	[Urinary dipsticks must not be used to detect diabetes-induced incipient nephropathy]	No outcome of interest
27	Lane	2006	Can spot urine protein/creatinine ratio replace 24 h urine protein in usual clinical nephrology?	No outcome of interest
28	Gai	2006	Comparison between 24-h proteinuria, urinary protein/creatinine ratio and dipstick test in patients with nephropathy: Patterns of proteinuria in dipstick-negative patients	No outcome of interest
29	Cortes-Sanabria	2006	Utility of the Dipstick Micral test II in the screening of microalbuminuria of diabetes mellitus type 2 and essential hypertension	No outcome of interest
30	Zeller	2005	Diagnostic significance of transferrinuria and albumin-specific dipstick testing in primary care patients with elevated office blood pressure	No outcome of interest
31	Naka	2005	Usefulness of protein/creatinine ratio in spot urine using test strips	No outcome of interest
32	Zeller	2005	Value of a standard urinary dipstick test for detecting microalbuminuria in patients with newly diagnosed hypertension	No outcome of interest
33	Hoy	2004	Albuminuria: marker or target in indigenous populations	No outcome of interest
34	Morishita	2004	Estimation of quantitative proteinuria using a new dipstick in random urine samples	No outcome of interest
35	Agarwal	2004	Quantitation of proteinuria by spot urine sampling	No outcome of interest
36	Parikh	2004	Rapid microalbuminuria screening in type 2 diabetes mellitus: Simplified approach with Micral test strips and specific gravity	No outcome of interest
37	Osta	2003	[Evaluation of two rapid tests for the determination of microalbuminuria and the urinary albumin/creatinine ratio]	No outcome of interest
38	Croal	2003	Evaluation of the Bayer Multistix PRO 10LS Point-of-Care Urine Test	No outcome of interest
39	Meinhardt	2003	Microalbuminuria in diabetes mellitus–Efficacy of a new screening method in comparison with timed overnight urine collection	No outcome of interest
40	Baskar	2003	Uncertain clinical utility of contemporary strategies for microalbuminuria testing	No outcome of interest
41	Agarwal	2002	Dipstick proteinuria: Can it guide hypertension management?	No outcome of interest
42	Wallace	2001	Multisite evaluation of a new dipstick for albumin, protein, and creatinine	No outcome of interest
43	Shihabi	2000	Clinical evaluation of a new strip test for proteinuria on ClinitekÂ® urinalysis systems	No outcome of interest
44	Lum	2000	How effective are screening tests for microalbuminuria in random urine specimens?	No outcome of interest
45	Davidson	1999	Relationship between dipstick positive proteinuria and albumin:creatinine ratios	No outcome of interest
46	Pugia	1999	Screening school children for albuminuria, proteinuria, and occult blood with dipsticks	No outcome of interest
47	Gerber	1998	Assessment of a new dipstick test in screening for microalbuminuria in patients with hypertension	No outcome of interest
48	Leong	1998	The use of semiquantitative urine test-strip (Micral-Test) for microalbuminuria screening in patients with diabetes mellitus	No outcome of interest
49	Minetti	1997	Accuracy of the urinary albumin titrator stick “Micral-Test” in kidney-disease patients	No outcome of interest
50	Pugia	1997	Comparison of urine dipsticks with quantitative methods for microalbuminuria	No outcome of interest
51	Gilbert	1997	Detection of microalbuminuria in diabetic patients by urinary dipstick	No outcome of interest
52	Adamson	1993	Screening strategies in the detection of microalbuminuria in insulin-dependent diabetic patients	No outcome of interest
53	Gilbert	1992	Semiquantitative determination of microalbuminuria by urinary dipstick	No outcome of interest
54	Kouri	1991	Microalbuminuria. Invalidity of simple concentration-based screening tests for early nephropathy due to urinary volumes of diabetic patients	No outcome of interest
55	Abitbol	1990	Quantitation of proteinuria with urinary protein/creatinine ratios and random testing with dipsticks in nephrotic children	No outcome of interest
56	Sawicki	1989	Comparison of Methods for Determination of Microalbuminuria in Diabetic Patients	No outcome of interest
57	Ralston	1988	Screening for proteinuria in a rheumatology clinic: comparison of dipstick testing, 24 h urine quantitative protein, and protein/creatinine ratio in random urine samples	No outcome of interest
58	Hemmingsen	1981	Diagnostic value of a test-strip in detecting increased urinary excretion of albumin, igg and Î^2^2-microglobulin in patients with suspected proteinuria	No outcome of interest
59	James	1978	Proteinuria: accuracy and precision of laboratory diagnosis by dipstick analysis	No outcome of interest
60	Delanghe	2017	Sensitive albuminuria analysis using dye-binding based test strips	No 24-hour urine albuminuria evaluation for the reference test
61	Asberg	2016	Using probit regression to disclose the analytical performance of qualitative and semiquantitative tests	No 24-hour urine albuminuria evaluation for the reference test
62	Turin	2014	Kidney function, albuminuria, and life expectancy	No 24-hour urine albuminuria evaluation for the reference test
63	Sarafidis	2008	A comparative evaluation of various methods for microalbuminuria screening	No 24-hour urine albuminuria evaluation for the reference test
64	Sam	2003	The significance of trace proteinuria	No 24-hour urine albuminuria evaluation for the reference test
65	Penders	2002	Quantitative evaluation of urinalysis test strips	No 24-hour urine albuminuria evaluation for the reference test
66	Pugia	2001	Albuminuria and proteinuria in hospitalized patients as measured by quantitative and dipstick methods	No 24-hour urine albuminuria evaluation for the reference test
67	Soonthornpun	2000	The Utility of Conventional Dipsticks for Urinary Protein for Screening of Microalbuminuria in Diabetic Patients	No 24-hour urine albuminuria evaluation for the reference test
68	Pegoraro	1997	Simplified screening for microalbuminuria	No 24-hour urine albuminuria evaluation for the reference test
69	Jensen	1996	The Micral test for diabetic microalbuminuria: predictive values as a function of prevalence	No 24-hour urine albuminuria evaluation for the reference test
70	Webb	1996	The use of the Micral-Test strip to identify the presence of microalbuminuria in people with insulin-dependent diabetes mellitus (IDDM) participating in the EUCLID study	No 24-hour urine albuminuria evaluation for the reference test
71	Bloomgarden	1996	Urine reagent stick protein determination: Utility in individuals with diabetes mellitus	No 24-hour urine albuminuria evaluation for the reference test
72	Gossain	1996	Utility of micral-test strips in screening for microalbuminuria	No 24-hour urine albuminuria evaluation for the reference test
73	de Grauw	1995	Screening for microalbuminuria in type 2 diabetic patients: the evaluation of a dipstick test in general practice	No 24-hour urine albuminuria evaluation for the reference test
74	Agardh	1993	A new semiquantitative rapid test for screening for microalbuminuria	No 24-hour urine albuminuria evaluation for the reference test
75	Tiu	1993	Comparison of six commercial techniques in the measurement of microalbuminuria in diabetic patients	No 24-hour urine albuminuria evaluation for the reference test
76	Jury	1992	Assessment of Micral-Test microalbuminuria test strip in the laboratory and in diabetic outpatients	No 24-hour urine albuminuria evaluation for the reference test
77	Marshall	1992	Micral-Test strips evaluated for screening for albuminuria	No 24-hour urine albuminuria evaluation for the reference test
78	Spooren	1992	Micral-Test: a qualitative dipstick test for micro-albuminuria	No 24-hour urine albuminuria evaluation for the reference test
79	Bangstad	1991	New semiquantitative dipstick test for microalbuminuria	No 24-hour urine albuminuria evaluation for the reference test
80	Oyebisi	2018	Prevalence and Pattern of Chronic Kidney Disease and its Associated Risk Factors in a Rural Community in South Western Nigeria	Full text not available
81	Ali	2017	Role of micral-test for the detection of microalbuminuria	Full text not available
82	Aziz	2015	Correlation of urine biomarkers: Microalbuminuria and spot urine protein among diabetic patients. Application of spot urine protein in diabetic kidney disease, nephropathy, proteinuria estimation, diagnosing, and monitoring	Full text not available
83	Shah	2015	Usefulness of spot urine protein creatinine ratio in the diagnosis of childhood nephrotic syndrome	Full text not available
84	Krairittichai	2011	Accuracy of urine dipstick test for microalbuminuria in type 2 diabetes mellitus patients	Full text not available
85	Chan	2005	Can the urine dipstick test reduce the need for microscopy for assessment of systemic lupus erythematosus disease activity?	Full text not available
86	Tsujikawa	2005	Evaluation of novel test strip to measure albumin and creatinine in urine	Full text not available
87	Le Floch	2001	Interest of clinitekÂ® microalbumin in screening for microalbuminuria: Results of a multicentre study in 302 diabetic patients	Full text not available
88	Ng	2000	Evaluation of a rapid screening test for microalbuminuria with a spot measurement of urine albumin-creatinine ratio	Full text not available
89	Ujjin	2000	Evaluation of microalb immunoturbidimetric test for albuminuria screening	Full text not available
90	Pugia	1998	Comparison of instrument-read dipsticks for albumin and creatinine in urine with visual results and quantitative methods	Full text not available
91	Fernandez	1998	Rapid screening test evaluation for microalbuminuria in diabetes mellitus	Full text not available
92	Jazayeri	1998	Urine protein dipstick measurements: A screen for a standard, 24-hour urine collection	Full text not available
93	Jensen	1993	Screening of microalbuminuria with the Micral-Test. A semiquantitative urinary dipstick	Full text not available
94	Schaufelberger	1992	[Evaluation of a strip test (Micral-test) for the semiquantitative assessment of microalbuminuria in clinical practice]	Full text not available
95	Poulsen	1992	Evaluation of a dipstick test for microalbuminuria in three different clinical settings, including the correlation with urinary albumin excretion rate	Full text not available
96	Allen	1991	Dipstick analysis of urinary protein. A comparison of Chempstrip-9 and Multistix-10SG	Full text not available
97	Coonrod	1989	Assessment of AlbuSure and its usefulness in identifying IDDM subjects at increased risk for developing clinical diabetic nephropathy	Full text not available
98	Poulsen	1995	Evaluation of a new semiquantitative stix for microalbuminuria	Letter to the editor
99	Dajak	2012	[Evaluation of methods for rapid microalbuminuria screening in kidney disease patients]	Serbian language

### 3.2 Study characteristics

Of the 14 included studies, five took place in Korea [22, 23, 24, 25, 26], four in the United Kingdom [8, 27, 28, 29], one in Italy [30], one in South Africa [31], one in Japan [32], one in Spain [33], and one in China [34]. Regarding the population, eight studies were performed in persons with comorbidities [8, 23, 24, 27, 28, 29, 31, 32], of which six were performed in persons with diabetes [23, 24,27, 28, 31, 32], one in persons with diabetes and/or CKD [29], and one in persons with CKD [8]. Furthermore, one study was performed in the general population [25], and one in general population or diabetic patients without CKD [30]; also, the remaining four studies did not define their study population [22, 26, 33, 34].

Regarding the assessed dipstick, the most used brand was Clinitek (Bayer and Siemens Medical Solutions Diagnostics) in six studies [24, 27, 29, 30, 31, 35] and was followed by URiSCAN (YD Diagnostics Corp.) in three studies [22, 23, 26]. Noting that each study used a different type of dipstick brand is important (Clinitek, Microalbustix, URiSCAN, Urisys, Uropaper, Siemens).

Thirteen out of 14 studies performed an automatic reading of the dipstick [22, 23, 24, 25, 26, 27, 29, 30, 31, 32, 33, 34, 35], and the remaining study performed a manual lecture [28]. Also, eight studies took a random urine sample [22 ,23 ,^25^, ^26^, ^29^, ^32^, ^34^, ^35^], four took an early morning sample [24, 27, 28, 30], and two did not define it [31, 33]. As for the reference standard, all studies have evaluated ACR, eight have collected random urine [22, 23, 25, 26, 29, 32, 34, 35], four have collected early morning sample [24, 27, 28, 30], and three did not define their sample [26, 31, 33] (Tables 3 and 4).

**Table 3:** Characteristics and risk of bias of the included studies.

Author (year)	Population/Setting	Sample size	Age	Dipstick that assessed ACR				Reference test (ACR)	Funding received	Risk of bias			
				Brand (Manufacturer)	Result	Lecture	Type of urine specimen	Type of urine specimen		Patient selection	Index test	Reference standard	Flow and timing
Parsons 1999 (UK) [29]	Diabetic and/or kidney failure patients/outpatient	144	Not defined	Clinitek (Bayer plc, Newbury)	Colors	Automatic	Random	Random	Industry	High	Unclear	High	Unclear
Croal 2001 (UK) [27]	Diabetic patients/outpatient	252	Not defined	Clinitek 50 (Bayer Diagnostics, Tarrytown, USA)	Not defined	Automatic	Early morning	Early morning	Government	High	Unclear	High	Unclear
Graziani 2009 (Italy) [30]	General population and diabetic patients/primary care	460	Not defined	Clinitek Microalbumin (Siemens Medical Solutions Diagnostics, Mishawaka, IN, USA)	Colors	Automatic	Early morning	Early morning	Industry	Low	Unclear	High	Low
Lloyd 2011 (South Africa) [31]	Diabetic Patients/outpatient	204	Not defined	Clinitek® (Siemens® Medical Solutions Diagnostics, formerly Bayer)	Not defined	Automatic	Not defined	Not defined	Industry	High	High	High	Low
Nagrebetsky 2013 (UK) [28]	Diabetic patients/outpatient	87	Mean: 68 yr	Microalbustix (Siemens Healthcare Diagnostics Ltd, Frimley, UK)	Colors	Manual	Early morning	Early morning	Government	High	Low	High	High
McTaggart 2012 (UK) [35]	Patients with or at risk of CKD/primary care	619	Mean: 66.5 yr	Clinitek Microalbumin 9 reagent strips (Siemens Medical Solutions Diagnostics)	Trace and +	Automatic	Random	Random	Industry	High	Low	High	Low
Cho 2014 (Korea) [22]	Not defined/not defined	1,040	Not defined	URiSCAN Super cassette ACR (YD Diagnostics Corp., Korea)	Trace and +	Automatic	Random	Random	Not mentioned	Unclear	Unclear	High	Low
Park 2017 (Korea) [25]	General population/National Survey	20,759	Mean: 46.6 yr	Urisys 2400 cassette strip (Roche, Mannheim, Germany)	Trace and +	Automatic	Random (early morning if possible)	Random (early morning if possible)	Government	Low	Unclear	High	High
Nah 2017 (Korea) [24]	Prediabetic (preDM) and diabetic (DM) patients/primary care	501	Median: 60 yr (preDM); 64 yr (DM)	Clinitek Microalbumin 2 reagent strips (Siemens, New York, NY, USA)	Colors	Automatic	Early morning	Early morning	Not mentioned	High	Unclear	High	Low
Lim 2018 (Korea) [26]	Not defined/not defined	1,020	Not defined	URiSCAN 2 ACR Strip (YD diagnostics, Yongin, Korea) and CLINITEK Microalbumin 2 Strip (Siemens, New York, NY, USA)	Trace and +	Automatic	Random	Random	Government	Unclear	Unclear	High	High
Shiwa 2018 (Japan) [32]	Diabetic patients/outpatient	291	Mean: 64.3 yr	Uropaper αIII (Eiken; Eiken Chemical, Tokyo, Japan)	Trace and +	Automatic	Random	Random	Not mentioned	High	Unclear	High	Low
Salinas 2018 (Spain) [33]	Not defined/primary care	9,148	Mean: 63 yr	Brand not defined (Sysmex, Kobe, Japan)	Not defined	Automatic	Not defined	Not defined	Not funded	High	Unclear	High	Unclear
Kim 2020 (Korea) [23]	Diabetic patients/outpatient	1,881 (development cohort); 431 (validation cohort)	Median: 66 yr (development cohort); 63 yr (validation cohort)	URiSCAN 2ACR strip (YD-Diagnostics Co., Yongin, Korea)	Colors	Automatic	Random	Random	Government	High	Unclear	High	Low
Yang 2020 (China) [34]	Not defined/not defined	1029	Not defined	Siemens Novus with Pro12 dipsticks (Amesdata Biotech Co.)	Not defined	Automatic	Random	Random	Not mentioned	Unclear	Unclear	High	Low

ACR: albumin-creatinine ratio; CKD: chronic kidney disease; preDM: prediabetes mellitus; DM: diabetes mellitus; yr: years; UK: the United Kingdom; +: expressed as 1+/2+/3+/4 + according to cutoff value.

**Table 4:** Study characteristics of individual studies.

Author (year)	Country	Population/Setting	Sample size	Age	Albuminuria prevalence (%)	Dipstick					Comparison				Outcome	Cutoff value	Funding
						Brand	Marker	Type of urine specimen	Result	Lecture	Laboratory test		Marker	Type of urine specimen			
											Albumin test	Creatinine test					
Parsons (1999) [29]	United Kingdom	Diabetic or kidney failure patients/outpatient	144	Not defined	55.56	Clinitek (Bayer plc, Newbury)	ACR	Random	Colors	Automatic (Cinitek-50, Bayer plc, Newbury)	Latex particle inmunoinhibition assay	Jaffe's method	ACR	Random	S, E, and PPV	30 mg/g	Grant from Bayer
Croal (2001) [27]	United Kingdom	Diabetic patients/outpatient	252	Not defined	20.63	Clinitek 50 (Bayer Diagnostics, Tarrytown, USA)	ACR	Early morning	Not defined	Automatic (Bayer Clinitek 50 urine chemistry analyzer, Bayer Diagnostics, Tarrytown, USA)	Nephelometry	Not defined	ACR	Early morning	S, E, PPV, and NPV	30 mg/g	Scottish Office Department of Health
Graziani (2009) [30]	Italy	General population and diabetic patients/primary care	460	Not defined	18.15	Clinitek Microalbumin (Siemens Medical Solutions Diagnostics, Mishawaka, IN, USA)	ACR	Early morning	Colors	Automatic (Clinitek Status, Siemens Medical Solutions Diagnostics, Mishawaka, IN, USA)	Nephelometry	Alkaline picrate method	ACR	Early morning	S, E, PPV, and NPV	30 and 300 mg/g	Strips and Instrument provided by Siemens
Lloyd (2011) [31]	South Africa	Diabetic patients/outpatient	204	Not defined	36.27	Clinitek®	ACR	Not defined	Not defined	Automatic (Clinitek® status reluctance photometer)	Immunone-phelometry	Jaffe's method	ACR	Not defined	S and E	3.4 mg/mmol (∼30 mg/g)	Instruments and reagents provided by Siemens and HemoCue
Nagrebetsky (2013) [28]	United Kingdom	Diabetic patients/outpatient	87	Mean: 68 yr	10.34	Microalbustix (Siemens Healthcare Diagnostics Ltd, Frimley, UK)	ACR	Early morning	Colors	Manually	Immunotur-bidimetry	Jaffe's method	ACR	Early morning	S and E	3.4 mg/mmol (∼30 mg/g)	National Institute for Health Research, United Kingdom
McTaggart (2012) [35]	United Kingdom	Patients with or at risk of CKD/primary care	619	Mean: 66.5 yr	20.19	CLINITEK Microalbumin 9 reagent strips	ACR	Random	Trace and +	Automatic (CLINITEK Status Analyzer)	Immunotur-bidimetry	Enzymatic methods	ACR	Random	S, E, PPV, NPV, and LR	30 mg/g	Grant by Siemens
Cho (2014) [22]	Korea	Not defined/not defined	1040	Not defined	47.12	URiSCAN Super cassette ACR	ACR	Random	Trace and +	Automatic (URiSCAN Super Plus, YD Electronics Co., Ltd., Korea)	Immunotur-bidimetry	Jaffe's method	ACR	Random	S, E, PPV, NPV, and correlation	30 and 300 mg/g	Not mentioned
Park (2017) [25]	Korea	General population/National Survey	20759	Mean: 46.6 yr	8.57	Urisys 2400 cassette strip (Roche, Mannheim, Germany)	ACR	Random (early morning if possible)	Trace and +	Automatic (Urisys 2400 automated analyzer, Roche, Mannheim, Germany)	Immunotur-bidimetry	Colorimetric assay and Jaffe's method	ACR	Random (early morning if possible)	S, E, PPV, and NPV	30 and 300 mg/g	Grant by Kangwon National University Hospital
Nah (2017) [24]	Korea	Prediabetic and diabetic patients/primary care	501	Median: 60 yr (preDM); 64 yr (DM)	21.96	CLINITEK Microalbumin 2 reagent strips (Siemens, New York, NY, USA)	ACR	Early morning	Colors	Automatic (CLINITEK Advantus Analyzer, Siemens)	Turbidimetric Immunoassay	Enzymatic methods	ACR	Early morning	S, E, PPV, and NPV	30 and 300 mg/g	Not mentioned
Lim (2018) [26]	Korea	Not defined/not defined	1020	Not defined	50.98 (URiSCAN); 49.24 (CLINITEK)	URiSCAN 2 ACR Strip and CLINITEK Microalbumin 2 Strip	ACR	Random	Trace and +	Automatic (URiSCAN New Pro, YD diagnostics, Yongin, Korea and Clinitek status plus, Siemens)	Immunoturb-idimetry	Jaffe's method	ACR	Random	S, E, PPV, NPV, and LR	30 and 300 mg/g	Ministry of Health & Welfare, Republic of Korea
Shiwa (2018) [32]	Japan	Diabetic patients/outpatient	291	Mean: 64.3 yr	29.55	Uropaper αIII (Eiken; Eiken Chemical, Tokyo, Japan)	ACR	Random	Trace and +	Automatic	Not defined	Not defined	ACR	Random	S, E, and ROC curve	30 and 300 mg/g	Not mentioned
Salinas (2018) [33]	Spain	Not defined/primary care	9148	Mean: 63 yr	13.98	Sysmex, Kobe, Japan	ACR	Not defined	Not defined	Automatic (UC-3500, Sysmex, Kobe, Japan)	Immunotur-bidimetry	Jaffe's method	ACR	Not defined	S, E, PPV, NPV, and LR	30 and 300 mg/g	Not funded
Kim (2020) [23]	Korea	Diabetic patients/outpatient	1881 (development cohort); 431 (validation cohort)	Median: 66 yr (development cohort); 63 yr (validation cohort)	42.53 (development cohort); 25.75 (validation cohort)	URiSCAN 2ACR strip	ACR	Random	Colors	Automatic (URiSCAN1 2ACR system, YD-Diagnostics Co., Yongin, Korea)	Immunotur-bidimetry	Jaffe's method	ACR	Random	S and E	30 and 300 mg/g	Ministry of Health & Welfare, Republic of Korea
Yang (2020) [34]	China	Not defined/not defined	1029	Not defined	50.94	Siemens Novus with Pro12 dipsticks	ACR	Random	Not defined	Automatic (Siemens Novus)	Immunotur-bidimetry	Not defined	ACR	Random	S, E, PPV, and NPV	30 and 300 mg/g	Not mentioned

ACR: albumin-creatinine ratio; preDM: prediabetes mellitus; DM: diabetes mellitus; +: expressed as 1+/2+/3+/4 + according to cutoff values; S: sensitivity; E: specificity; PPV: positive predictive value; NPV: negative predictive value; ROC: receiver operating characteristic; LR: likelihood ratio.

### 3.3 Results of meta-analyses

**Figure 2:**
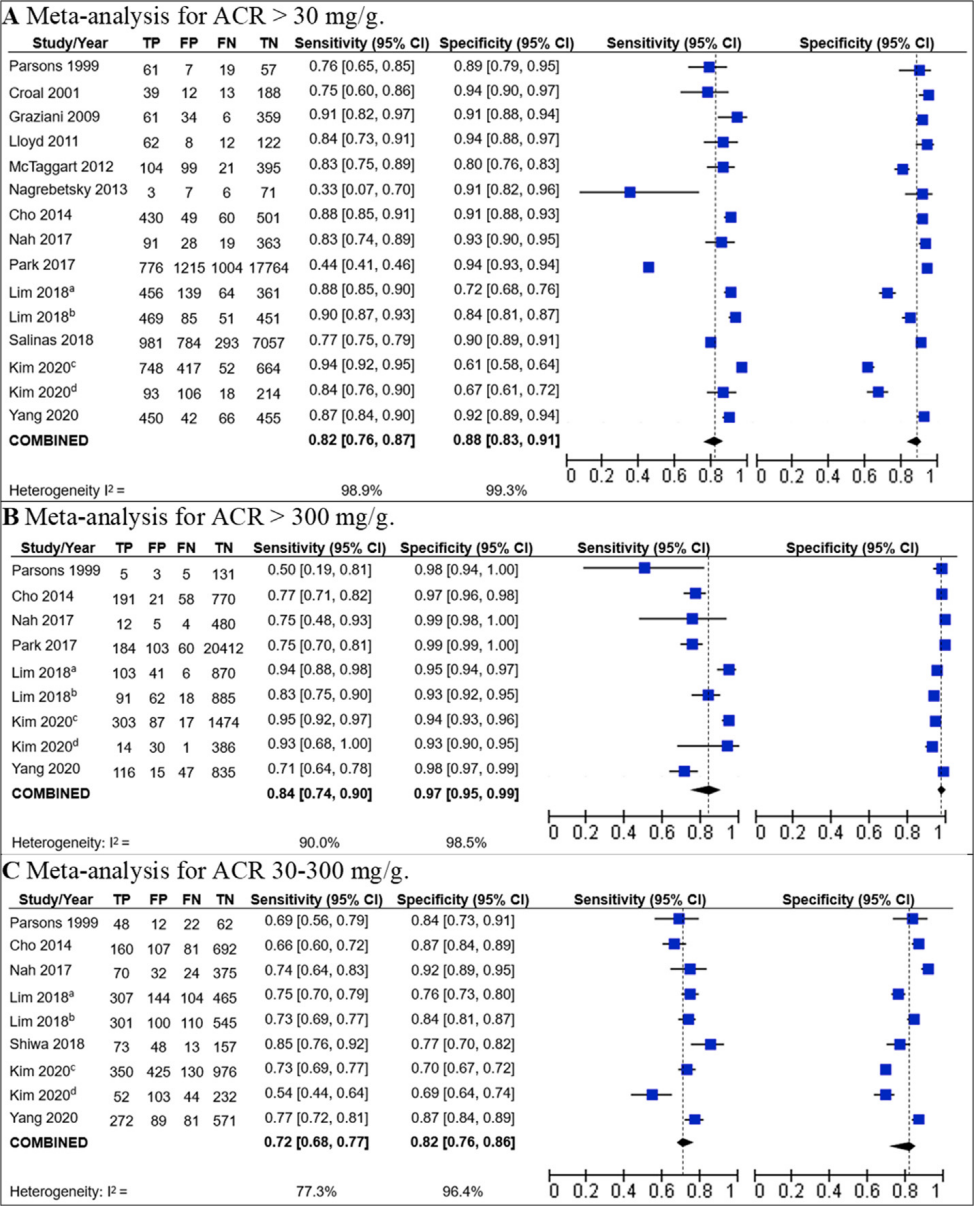
Forest plots on sensitivity and specificity for urine dipstick testing ACR. ^a^This observation corresponds to the results for the URiSCAN 2 ACR Strip dipstick brand reported by Lim 2018. ^b^This observation corresponds to the results for the CLINITEK Microalbumin 2 Strip dipstick brand reported by Lim 2018. ^c^This observation corresponds to the “development cohort” from Kim 2020. ^d^This observation corresponds to the “validation cohort” from Kim 2020.

Twelve studies were included for the meta-analyses that assessed dipstick accuracy for the cutoff point of ACR >30 mg/g [22, 23, 24, 25, 26, 27, 29, 30, 31, 33, 34, 35], and the pooled estimate gave a sensitivity and specificity of 0.82 (95% CI 0.76–0.87) and 0.88 (95% CI 0.83–0.91), respectively. Regarding the cutoff point of ACR 30–300 mg/g, seven studies were included [22, 23, 24, 26, 29, 32, 34], and the pooled estimate gave a sensitivity and specificity of 0.72 (95% CI 0.68–0.77) and 0.82 (95% CI 0.76–0.86), respectively. Regarding the cutoff point of ACR >300 mg/g, seven studies were included [22, 23, 24, 25, 26, 29, 34], and the pooled estimate gave a sensitivity and specificity of 0.84 (95% CI 0.74–0.90) and 0.97 (95% CI 0.95–0.99) (Figure 2).

Kim 2020 is mentioned twice in the meta-analyses: one for its developmental cohort and the other for its validation cohort [23]. Also, Lim 2018 is mentioned twice: one for each dipstick used, one for the use of CLINITEK Microalbumin 2 Strip dipstick brand, and the other for the use of URiSCAN 2 ACR Strip dipstick brand [26].

All of these meta-analyses showed high heterogeneity (I^2^ being higher than 70% for all of the meta-analyses). For the dipstick's sensitivity for ACR >30 mg/g, heterogeneity was found to be mainly generated by two studies: Nagrebetsky 2013 and Park 2017. Nagrebetsky 2013 is the only study that used a manual lecture of the dipstick result, which may explain its low sensitivity [28]. However, the study by Park 2017 does not have a special characteristic that could explain the lower sensitivity with respect to the rest of studies, although it was the only study that used the Urisys 2400 cassette strip recorded from a device (Urisys 2400 automated analyzer, Roche, Mannheim, Germany) for the diagnosis of albuminuria [25].

A sensitivity analyses have been performed excluding the only study that used a manual lecture of the dipstick (Nagrebetsky 2013), for the ACR >30 mg/g cutoff point. The resulting sensitivity and specificity were: 0.83 (95% CI: 0.78–0.88, I^2^ = 99%) and 0.87 (95% CI: 0.82–0.91, I^2^ = 99%).

### 3.4 Risk of bias

In the patient selection domain, most of the studies (9/14) had a high risk of bias, and 3/14 had unclear risk bias. The most frequent limitation was that studies did not avoid inappropriate exclusion. For the index test domain, most of the studies were considered to have an unclear risk of bias (11/14) due to a lack of information regarding the interpretation of the index test. For the reference standard domain, all studies were found to have a high risk of bias because the reference tests used were not the gold standard (turbidimetry, nephelometry, or radioimmunoassay for albuminuria; isotope dilution mass spectrometry for creatinine), and whether the interpretation of the results of the index and reference tests was independent was not clear. With regard to the flow and timing domain, 3/14 studies were found to have a high risk of bias and 3/14 had an unclear risk of bias because not all patients were included in the analysis or different standard tests were used. Additionally, the intervals between the index test and the reference standard were not clear for some studies (Table 3).

### 3.5 Summary of findings

A Summary of Findings (SoF) table has been performed. All the pooled sensitivities and specificities had a very low certainty of the evidence, due to a high or very high risk of bias, inconsistency, and imprecision.

To estimate the impact that the pooled sensitivity and specificity would have as regards false-positive and false-negative cases, these were estimated a fictitious population of 1,000 individuals. For these estimations, different prevalences of ACR >30 mg/g, between 30 mg/g and 300 mg/g, and >300 mg/g were assumed and calculated using the median prevalence of studies that showed information for the prevalence of all these three cutoff values [22, 23, 24, 26, 29, 34] (Table 5).

**Table 5:** Summary of findings to evaluate the certainty of the evidence, using the GRADE methodology.

Cutoff point	Number of studies (participants)	Summary of sensitivity (95% CI)	Summary of specificity (95% CI)	Certainty of the evidence^b^	Consequences in a fictitious population of 1000 patients^a^		
					Assumed prevalence	Underdiagnosed or false negatives (95% CI)	Overdiagnosed or false positives (95% CI)
ACR >30 mg/g	13 (38,582)	0.82 (0.76–0.87)	0.88 (0.83–0.91)	Sensitivity: very low^1,2,3^ Specificity: very low^1,2^	48.2%	87 (63 a 116)	62 (47 a 88)
ACR 30–300 mg/g	7 (7,377)	0.72 (0.68–0.77)	0.82 (0.76–0.86)	Sensitivity: very low^1,4^ Specificity: very low^1,2^	30.2%	85 (69 a 97)	126 (98 a 168)
ACR >300 mg/g	7 (27,596)	0.84 (0.74–0.90)	0.97 (0.95–0.99)	Sensitivity: very low^1,2^ Specificity: very low^1,2^	10.5%	17 (11 a 27)	27 (9 a 45)

ACR: albumin-creatinine ratio; CI: confidence intervals.

a This fictitious population is assumed to have a prevalence of ACR >30 mg/g of 48.2 %, a prevalence of ACR between 30 mg/g and 300 mg/g of 30.2%, and a prevalence of ACR >300 mg/g of 10.5%. Median of the prevalence of studies that have reported ACR cutoff point >30, 30–300, and >300 mg/g (Parsons et al., Cho et al., Nah et al., Lim et al., Kim et al. and Yang et al.).

b Explanation of the certainty of the evidence: 1. Very high risk of bias, 2. very high inconsistency, 3. high imprecision, 4. high inconsistency.

## 4 Discussion

### 4.1 Comparison with previous studies

One previous systematic review was found that assessed the diagnostic accuracy of POC tests (either semiquantitative or quantitative) in people at risk of CKD. It performed its literature search in December 2013 and meta-analyzed nine studies that assessed urine dipsticks, obtaining a pooled sensitivity and specificity of 0.76 and 0.93, respectively, for ACR >30 mg/g [8]. Five of the nine studies included by such review have been included in our study. The other four studies were excluded since two of them evaluated cutoff values different than ours [36, 37], one did not have its full text available [38], and one was published as a letter to the editor [39].

Moreover, other eight studies have been included that assessed ACR >30 mg/g, for a total of 13 meta-analyzed studies, finding that a urine dipstick test has a sensitivity and specificity of 0.82 (95% CI: 0.76–0.87) and 0.88 (95% CI: 0.83–0.91), respectively; while the cited systematic review found a sensitivity and specificity of 0.76 (95% CI: 0.63–0.86) and 0.93 (95% CI: 0.84–0.97), respectively. As seen, confidence intervals overlapped considerably. Furthermore, while the cited systematic review reported their result uniquely for the ACR >30 mg/g cutoff value, the ACR between 30 mg/g and 300 mg/g and the ACR >300 mg/g have also been included due to their clinical relevance.

### 4.2 Implications for clinical practice

Urine dipsticks represent a time-saving POC screening test in resource-limited settings that lack laboratory analysis [40]. However, to adequately decide whether to use urine dipsticks, or in cases which use it, stakeholders should consider its sensitivity and specificity, along with the expected number of false positives and false negatives for their study population.

For detecting ACR >30 mg/g, dipsticks showed a limited sensitivity (0.82, 95% CI: 0.76–0.87) and specificity (0.88, 95% CI: 0.83–0.91). A suboptimal accuracy could be expected, due to the effect of some interfering compounds (drugs, vitamins, urine preservatives, and detergents), urine pH, and storage deficiencies [40].

As shown in the SoF table, for a 1,000-patient population with a prevalence of ACR >30 mg/g of 48.2%, the pooled accuracy was translated into 87 false-negative results and 62 false-positive results. Each one of these groups has a different impact. Since CKD Clinical guidelines recommend that patients with positive dipstick results are assessed using quantitative methods before establishing the CKD diagnosis [1, 2], false positives could be corrected in this step, although causing preoccupation and expenses for these patients. Conversely, false-negative results could be more problematic, since these cases could mean a loss of opportunity for performing a timely diagnosis and management of CKD. Repeated dipstick assessments could diminish the false-negatives rates. However, KDIGO and NICE guidelines do not state any recommendation regarding the frequency of ACR testing. However, the Australian CKD guidelines recommend annual screening of urine ACR in people at risk of CKD [41].

### 4.3 Impact in progression

The detection of ACR >300 mg/g is also relevant in assessing CKD progression. In CKD patients, the KDIGO guideline recommends annual albuminuria tests or every 1–3 months in patients with a high risk of CKD progression [1]. Furthermore, the NICE guidelines establish a frequency of monitoring the glomerular filtration rate (GFR) per year depending on ACR and GFR in people with or at risk of CKD [2]. For a population of 1,000 persons with an ACR >300 mg/g prevalence of 10.5% (i.e., 105 persons), the use of dipsticks has been estimated to end in 17 false negatives and 27 false positives. Analyzing how repeated dipstick assessment yielded negative results remains relevant, and confirmation with quantitative methods in positive ones would improve these results.

Performing economic analyses that could guide decision-making regarding urine dipsticks’ use for a specific country or region is also important. These analyses should perform sensitivity analyses for different types of patients, with different frequencies of dipstick use, and with different decision trees. Only one economic analysis performed for Korean diabetic patients with a GFR >60 ml/min per 1.73 m^2^ and a negative urine dipstick result has been found. For this analysis, the authors considered a sensitivity and specificity of 0.84 and 0.67, respectively, for ACR >30 mg/g, values from one of the studies included in our systematic review [23]. In this model, testing the annual progression with a semiquantitative tool saved 16.7% (339.6 USD) of costs per diabetic patient for 10 years compared with doing it with a quantitative test [23].

### 4.4 Limitations and strengths

This study has some limitations that must be considered to adequately interpret our results: 1) Subgroup analyses according to the operator (laboratory vs. clinical) were not performed due to the lack of precise data on this variable given in the articles, according to the dipstick brand due to the high variability, or according to the lecture (manually/automatic) since only one study used manual lecture. 2) Sensitivity and specificity may improve after indexing dipstick results for urine concentration [42, 43]; however, dipstick results have not been indexed due to a lack of reporting data in the included studies. 3) ACR results were not adjusted by muscle mass, and albuminuria reference tests are not well standardized in the included studies, a flaw that may guide to obtain imprecise results. 4) Our results had a very low certainty of the evidence, so future well-designed studies are needed to reach a conclusion and to explain the heterogeneity found in our results. 5) The accuracy of different dipstick brands and types could not be assessed since each study used a different type of dipstick.

However, this study considered a broad inclusion criteria for the population (general population and people at risk or with CKD). Furthermore, a meta-analyses for the three cutoff values (ACR >30, 30–300, >300 mg/g) have been performed with clinical relevance according to current clinical guidelines.

## 5 Conclusion

A total of 14 studies were found and assessed for sensitivity and specificity of urine dipsticks for ACR assessment, and no study was found that assessed urine dipsticks for albuminuria assessment. Dipstick types used were different across studies. The sensitivity and specificity of dipsticks have been pooled for ACR >30 mg/g, ACR 30–300 mg/g, and ACR >300 mg/g. However, all meta-analyses showed high heterogeneity, and the certainty of the evidence was very low for all the results. Thus, further well-designed studies are needed to reach more confident estimates and to assess accuracy differences across dipstick types. Until then, clinical practitioners should be cautious when interpreting dipstick results.

## Declarations

### Author contribution statement

Jhonatan R. Mejia, Jose Ernesto Fernandez-Chinguel: Conceived and designed the experiments; Performed the experiments; Analyzed and interpreted the data; Contributed reagents, materials, analysis tools or data; Wrote the paper.

Gandy Dolores-Maldonado, Naysha Becerra-Chauca, Sergio Goicochea-Lugo: Performed the experiments; Contributed reagents, materials, analysis tools or data; Wrote the paper.

Percy Herrera-Añazco: Analyzed and interpreted the data; Contributed reagents, materials, analysis tools or data; Wrote the paper.

Jessica Hanae ZafraTanaka, Alvaro Taype-Rondan: Conceived and designed the experiments; Analyzed and interpreted the data; Contributed reagents, materials, analysis tools or data; Wrote the paper.

### Funding statement

This research did not receive any specific grant from funding agencies in the public, commercial, or not-for-profit sectors.

### Data availability statement

The protocol is registered at PROSPERO (CRD42019124637) available at https://www.crd.york.ac.uk/prospero/display_record.php?RecordID=124637. The datasets used and/or analyzed during the current study are available at dx.doi.org/10.6084/m9.figshare.13350923.

### Declaration of interests statement

The authors declare no conflict of interest.

### Additional information

No additional information is available for this paper.
